# Case Report: Trastuzumab deruxtecan in human epidermal growth factor receptor 2-mutated lung cancer with continuous renal replacement therapy

**DOI:** 10.3389/fonc.2025.1722169

**Published:** 2026-01-07

**Authors:** Sumin Wu, Chengming Ke, Min Wei

**Affiliations:** 1 Center of Excellence, The Seventh Affiliated Hospital, Sun Yat-sen University, Shenzhen, Guangdong, China; 2 Neuroscience Medical Center, The Seventh Affiliated Hospital, Sun Yat-sen University, Shenzhen, Guangdong, China

**Keywords:** HER2-mutated lung cancer, T-DXd, CRRT, ADC, AKI

## Abstract

**Background:**

Human epidermal growth factor receptor 2 (HER2)-mutated lung cancer is a rare and aggressive subtype of non-small cell lung cancer (NSCLC), characterized by poor prognosis and limited response to conventional therapies. Trastuzumab deruxtecan (T-DXd), a HER2-targeting antibody-drug conjugate (ADC), has shown promising results in HER2-mutated cancers. However, its safety and efficacy in patients with renal dysfunction requiring continuous renal replacement therapy (CRRT) remain unclear.

**Case summary:**

A 69-year-old female with advanced HER2-mutant NSCLC developed acute kidney injury (AKI) requiring intermittent CRRT after failing standard chemotherapy. With strong patient commitment, T-DXd was initiated following multidisciplinary discussion. Although severe myelosuppression occurred following targeted therapy, it resolved with appropriate supportive care. Notably, no significant toxicities such as interstitial lung disease, hepatotoxicity, or further nephrotoxicity were observed. After three cycles of T-DXd, symptomatic improvement was achieved, including resolution of abdominal distension, significant reduction of ascites, and disappearance of hematuria. Follow-up imaging studies confirmed stable disease. Unfortunately, the patient succumbed to aspiration, which precluded administration of further T-DXd cycles.

**Conclusion:**

T-DXd treatment in this CRRT-dependent patient with HER2-mutant lung cancer achieved disease control with manageable toxicity. While demonstrating potential clinical utility, the short survival period warrants cautious interpretation. Further validation is needed to establish its role in this population.

## Introduction

Human epidermal growth factor receptor 2 (HER2) mutations have been increasingly recognized as a critical driver of malignancy in non-small cell lung cancer (NSCLC) (1), particularly in patients with advanced or metastatic disease. HER2-mutant lung cancer is known for its aggressive clinical course, poor prognosis, and resistance to conventional therapies, including chemotherapy and immunotherapy (2). Targeted therapies designed to inhibit HER2, such as trastuzumab deruxtecan (T-DXd), have demonstrated efficacy in other cancers, including breast cancer (3). T-DXd is now the recommended second-line therapy for HER2-mutant NSCLC per NCCN and other international guidelines, following platinum-based chemotherapy (4, 5).

Continuous Renal Replacement Therapy (CRRT) is primarily used in cancer patients to manage critical conditions complicated by acute kidney injury (AKI), particularly tumor lysis syndrome, septic shock, or anticancer drug-related nephrotoxicity. Compared to intermittent hemodialysis, CRRT offers slower and sustained solute clearance and ultrafiltration, which enables better hemodynamic stability, precise fluid balance control, and effective removal of inflammatory mediators and certain chemotherapeutic agents (6). It provides continuous support for renal function, allowing for the removal of waste products, fluid management, and the correction of electrolyte imbalances (7). However, its use in cancer patients, particularly those undergoing targeted therapy, has been less well-documented. This case explores the combination of T-DXd with CRRT in a patient with advanced HER2-mutant lung cancer, highlighting the potential clinical significance and safety of this approach.

## Case presentation

**Figure 1:**
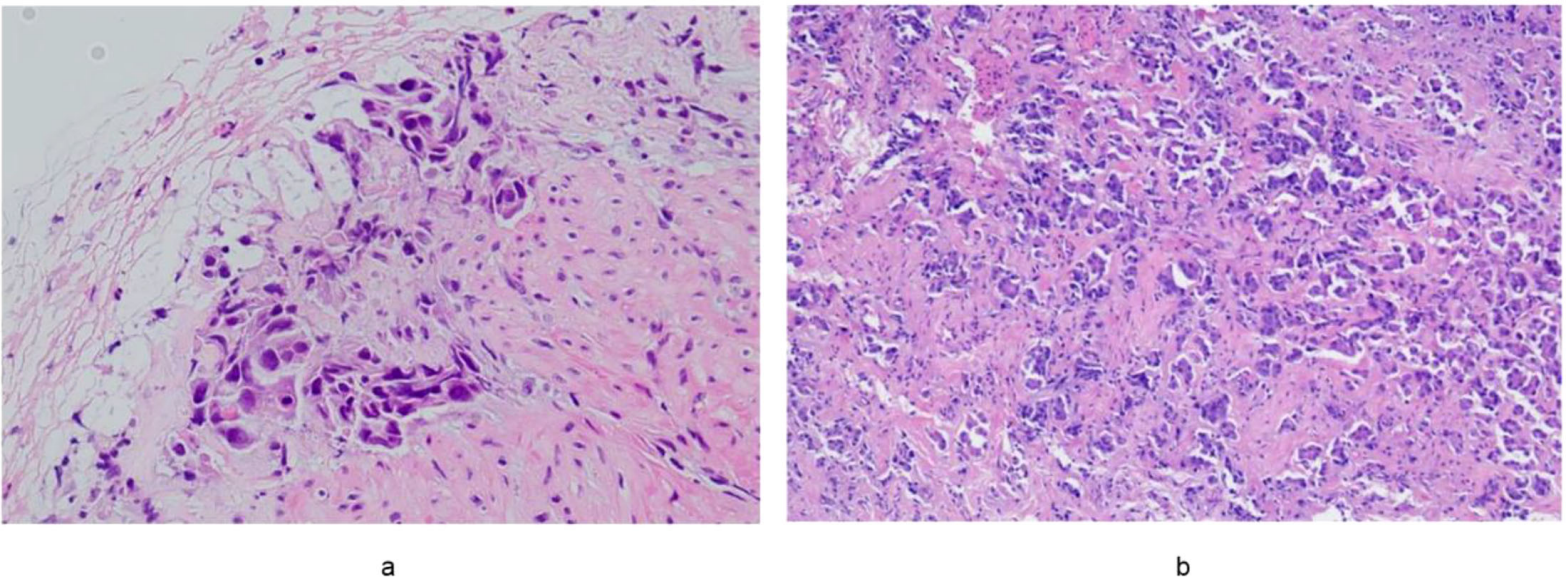
**(a)** Hematoxylin and eosin staining (HE×20) of a biopsy specimen obtained by gastroscopy revealing lung-derived esophageal metastasis. **(b)** Hematoxylin and eosin staining (HE×20) of a biopsy specimen obtained by cystoscopy revealing lung-derived bladder metastasis.

The patient was a 69-year-old woman with a history of hypertension for over 10 years. In March 2024, she presented to a local hospital with a one-month history of recurrent cough and dyspnea. Physical examination revealed diminished breath sounds in the right lung. Chest CT imaging identified a large right pleural effusion. Thoracentesis and drainage were performed, and cytological analysis of the pleural fluid revealed numerous atypical cells. Whole-body PET/CT demonstrated focal thickening of the mid-to-distal esophageal wall, along with multiple FDG-avid enlarged lymph nodes in the bilateral supraclavicular regions, right axilla, medial to the right pectoralis minor muscle, bilateral hilar and mediastinal areas, parasternal region, right anterior costophrenic angle, gastrohepatic space, and para-aortic regions, highly suggestive of esophageal carcinoma with lymph node metastasis. Gastroscopy confirmed focal thickening in the lower thoracic esophagus, while colonoscopy was unremarkable. Endoscopic ultrasound-guided biopsy of the esophageal lesion revealed fibrous connective and smooth muscle tissue infiltrated by clusters and scattered individual cells with enlarged, eccentrically located nuclei. The histopathological diagnosis was malignant tumor, consistent with invasive adenocarcinoma. Based on imaging and immunohistochemical findings, a pulmonary origin was considered most likely (**Figure 1a**). Immunohistochemistry results were as follows: P40 (–), CK7(+), TTF-1(+), Napsin A(–), ALK (D5F3)(–), ALK-N(–), CEA(–), Villin(–), STAB2(–), GATA-3 (focally+), CK5/6(–), CDX2 (rare weakly+).Next-generation sequencing (NGS) identified an ERBB2 exon 19 p.L755P missense mutation (variant allele frequency: 15.82%), a TP53 mutation, and MYC gene amplification. The final diagnosis was stage IV poorly differentiated HER2-mutant lung adenocarcinoma.

From April 15 to July 30, 2024, the patient received first-line therapy with six cycles of pemetrexed, carboplatin, pembrolizumab, and bevacizumab. During treatment, her serum creatinine rose from normal to 102.8 μmol/L(1.16mg/dL), necessitating dose reduction of the chemotherapeutic agents. On August 20, 2024, she developed abdominal distension and gross hematuria. Follow-up CT imaging indicated disease progression, prompting a change in regimen to albumin-bound paclitaxel, cisplatin, pembrolizumab, and bevacizumab. Her symptoms progressively worsened, accompanied by anorexia and postprandial vomiting.

She was transferred to our institution for further management on November 6, 2024. Physical examination revealed pale skin and mucous membranes, reduced breath sounds on the right, marked abdominal distension, and shifting dullness. Laboratory studies showed hemoglobin at 86 g/L and serum creatinine elevated to 210.24 μmol/L(2.38mg/dL). Whole-body contrast-enhanced CT revealed disease progression with new findings including multiple right lung lesions, numerous hepatic metastases, diffuse gastric wall thickening, diffuse bladder wall thickening, thickening of the left ureter’s pelvic segment with associated left hydronephrosis, peritoneal thickening and extensive ascites. Pathological assessment of a bladder biopsy confirmed metastatic lung adenocarcinoma (**Figure 1b**). Immunohistochemistry of the tumor cells showed TTF-1(+), ER(–), GATA3(–), SOX-10(–), and Mammaglobin(–).During hospitalization, she underwent drainage of right pleural effusion and ascites, along with supportive care. On November 16, 2024, she developed oliguria and generalized edema. Blood tests revealed hyperkalemia, metabolic acidosis, and a progressive rise in serum creatinine to 616.42 μmol/L(6.97mg/dL). CRRT in Continuous Veno-Venous Hemofiltration (CVVH) mode was initiated on November 18, 2024, for life support. Despite AKI, after thorough discussion and respecting the patient’s strong preference, targeted therapy with T-DXd at 3.2 mg/kg was administered on November 21, 2024. The second and third cycles of T-DXd were given on December 20, 2024, and January 18, 2025, respectively.

**Figure 2:**
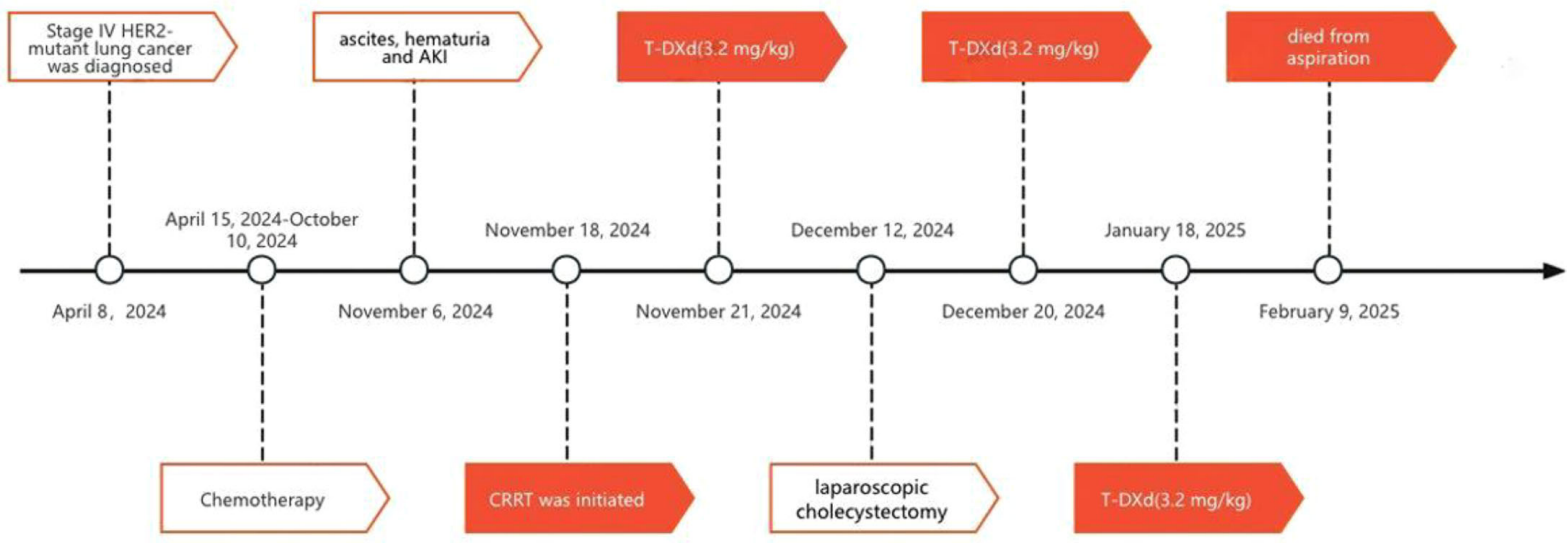
Flow chart of patient from diagnosis to current treatment. HER2, human epidermal growth factor receptor 2; AKI, acute kidney injury; CRRT, continuous renal replacement therapy; T-DXd, trastuzumab deruxtecan.

Treatment-related toxicities were manageable. Hematologic adverse events included grade III neutropenia and thrombocytopenia following targeted therapy, both resolving with supportive measures. Significant hepatic or pulmonary toxicity was not observed. Regarding renal function, CRRT was discontinued on November 28, 2024, following clinical improvement characterized by urine output exceeding 1000 mL/day, decline in serum creatinine to approximately 250 μmol/L(2.82mg/dL), and stabilization of her internal environment. However, due to a subsequent rebound in creatinine, CRRT was resumed on January 17, 2025. Notably, on December 12, 2024, prior to the second T-DXd cycle, she underwent laparoscopic cholecystectomy for cholecystitis; pathology confirmed metastatic lung adenocarcinoma involving the gallbladder wall. After three cycles of T-DXd treatment, her clinical condition improved, with alleviated abdominal distension, significantly reduced ascites, and resolved hematuria. A follow-up CT scan on January 20, 2025, showed stable disease with minimal regression in some mediastinal, bilateral hilar, and axillary lymph nodes, as well as hepatic metastases. Unfortunately, the patient died due to aspiration on February 9, 2025 (**Figure 2**).

## Discussion

HER2-mutant lung cancer demonstrates a more aggressive clinical course and distinct metastatic behavior (8). The metastatic spread in this case was extensive. HER2-mutant lung cancer is generally poorly responsive to conventional chemotherapy and immunotherapy (9), as observed in this patient. T-DXd has been shown to be effective in treating adult patients with unresectable locally advanced or metastatic NSCLC harboring HER2 (ERBB2) activating mutations (5, 10). T-DXd is an antibody-drug conjugate (ADC) targeting HER2. It consists of a humanized anti-HER2 immunoglobulin G1 (IgG1) monoclonal antibody linked to the topoisomerase I inhibitor DXd. Upon binding to HER2 receptors on tumor cells, T-DXd undergoes internalization, and its linker is cleaved by lysosomal enzymes. The released DXd induces DNA damage, leading to tumor cell apoptosis (4). Pharmacokinetic studies have shown that DXd is primarily metabolized by CYP3A4 in the liver and excreted via bile, with most of the drug appearing in urine, feces, and bile (5). The pharmacokinetics of T-DXd or its released DXd are not affected by mild to moderate renal impairment (11). However, caution is advised in patients with severe renal impairment or undergoing dialysis, as there are no studies in these populations (12).

In this case, T-DXd was initiated during continuous renal replacement therapy (CRRT) for acute kidney injury (AKI). Nephrology consultation confirmed that the AKI was multifactorial in origin, attributable to renal hypoperfusion from inadequate intake and third-space fluid sequestration, nephrotoxic effects of prior chemotherapy (particularly platinum-based agents), and obstructive uropathy secondary to bladder metastasis with associated left hydronephrosis. Although severe myelosuppression occurred following targeted therapy, it resolved with appropriate supportive care. Notably, no significant toxicities such as interstitial lung disease, hepatotoxicity, or further nephrotoxicity were observed. During the treatment course, the patient also successfully underwent cholecystectomy. After three cycles of T-DXd, symptomatic improvement was achieved, including resolution of abdominal distension, significant reduction of ascites, and disappearance of hematuria. Follow-up imaging studies confirmed stable disease. Together, these findings suggest meaningful clinical benefit from T-DXd in this complex clinical scenario. Nonetheless, the patient’s short overall survival period necessitates cautious interpretation of these findings, and further investigations are required to validate these observations.

## Data Availability

The raw data supporting the conclusions of this article will be made available by the authors, without undue reservation.
